# Positron Emission Tomography Imaging of Macrophages in Cancer

**DOI:** 10.3390/cancers13081921

**Published:** 2021-04-16

**Authors:** Candace C. Parker, Suzanne E. Lapi

**Affiliations:** Departments of Chemistry and Radiology, University of Alabama at Birmingham, Birmingham, AL 35294, USA; parker6@uab.edu

**Keywords:** PET, imaging biomarkers, radiotracers, macrophages, TAMs

## Abstract

**Simple Summary:**

Tumor-associated macrophages (TAMs) play numerous roles in cancer biology and are an important component of the relationship between immune system response and tumor progression. Several new immunotherapy techniques have been developed that target TAMs and are under investigation in both clinical and preclinical settings. Despite this surge of new immunotherapy techniques, a means to specifically and quantifiably measure the presence of TAMs to ensure the viability of these therapies, has yet to be widely investigated. The development of molecular imaging agents that target TAMs provides a path to noninvasively gain valuable insight into the molecular and functional characteristics of the tumor microenvironment and how the immune response facilitates the progression of cancer or therapy response. This article reviews published preclinical and clinical research in the imaging of TAMs through Positron Emission Tomography (PET).

**Abstract:**

Macrophages are large phagocytic cells that can be classified as a type of white blood cell and may be either mobile or stationary in tissues. The presence of macrophages in essentially every major disease makes them attractive candidates to serve as therapeutic targets and diagnostic biomarkers. Macrophages that are found in the microenvironment of solid tumors are referred to as tumor-associated macrophages (TAMs) and have been shown to influence chemoresistance, immune regulation, tumor initiation and tumor growth. The imaging of TAMs through Positron Emission Tomography (PET) has the potential to provide valuable information on cancer biology, tumor progression, and response to therapy. This review will highlight the versatility of macrophage imaging in cancer through the use of PET.

## 1. Introduction

The year 1883 marked the beginning of an exciting new era for immunology. Ellie Metchnikoff introduced the concept of macrophages by determining phagocytes functions as a critical host-defense mechanism. This eventually led Metchnikoff and Paul Ehrlich to receive a Nobel Prize for their work on immunity in 1908. This study provided the earliest description of macrophages and opened up a new field of research as numerous scientists propelled the field forward by working to advance current knowledge and understanding of the role of macrophages in many disease states [1,2].

Today, macrophages are described as large phagocytic cells that can be classified as a type of white blood cell that may be either mobile or stationary in tissues [3]. They are involved in a variety of significant roles and functions such as wound healing facilitation, tissue homeostasis maintenance, pathogen defense, immune system stimulation, and foreign substance digestion [3].

Macrophages represent a diverse set of cells which are associated with a distinct set of disease states such as tuberculosis [4], sepsis [5], neurodegenerative disease [6,7], infection [8], chronic inflammatory diseases [9], and cancer [10,11,12,13]. The presence of macrophages in essentially every major disease makes them attractive candidates to serve as therapeutic targets and diagnostic biomarkers. Macrophages that are found in the microenvironment of solid tumors are referred to as tumor-associated macrophages (TAMs). They have been shown to influence chemoresistance, immune regulation, tumor initiation, and tumor growth [10]. Macrophages can directly promote tumor growth through angiogenesis and resistance to certain types of chemotherapies. Indirect promotion of tumor growth can also be influenced as macrophages can induce immune dysfunctions through interactions with other immune cells within the tumor microenvironment [14].

Macrophages can be broadly classified into two main groups with differing roles in immune defense and immune surveillance: classically activated macrophages (M1) and alternatively activated macrophages (M2) [1,3]. M1 and M2 represent polarization states that result from differentiation by the local microenvironment. M1 macrophages are characterized by possessing pro-inflammatory and microbicidal functions whereas M2 macrophages prevent inflammation and promote tissue remodeling and angiogenesis [15]. M1 macrophages can be considered antitumor due to their ability to kill tumor cells through the production of pro-inflammatory cytokines. As a result, M1 polarized macrophages can be thought of as effector cells that possess the important role of protecting the body from pathogen and tumor cell attacks [16]. M2 macrophages can be considered protumor due to their production of anti-inflammatory cytokines [11].

Macrophage polarization can be influenced by antitumoral treatments such as chemotherapy and radiotherapy and thus can facilitate resistance to such therapies [17,18]. Immunotherapy treatments can also be negatively affected by the presence of TAMs [19]. In particular, PD-L1 expression on TAMS have been shown to diminish the therapeutic response of PD-1 targeted therapies such as Pembrolizumab (Keytruda) and Nivolumab (Opdivo) [19,20].

It has previously been demonstrated that the presence of TAMs in a variety of human cancers can result in a poor clinical prognosis. Such examples include gastric cancer [21], colorectal cancer [22], prostate cancer [23], and Classical Hodgkin lymphoma [24]. An extensive list can be found in the literature review of Baojin Hua and colleagues [12].

TAMs have become increasingly more relevant in cancer biology as they represent a biomarker that could lead to improved diagnostic and prognostic outcomes [25]. The understanding of the regulation of the immune system response to tumor progression by TAMs has resulted in the development of several therapies that target TAMs in the preclinical and clinical settings [26]. Thus, a means to specifically and quantifiably measure the presences of the TAMs, preferably in a non-invasive manner, is an important tool to ensure the viability of these therapies.

Despite this need, clinical imaging of tumor-associated macrophages has yet to be widely investigated. The development of molecular imaging agents that specifically target TAMs is an area that is growing in order to address this need. The imaging of macrophages can provide a noninvasive approach to gain valuable insight into the molecular and functional characteristics of the tumor microenvironment and how the immune response facilitates the progression of cancer or therapy response.

Positron emission tomography (PET) is a form of molecular imaging that allows for a noninvasive assessment of physiologic information by utilizing radioactive contrast agents. It is widely used in both clinical and preclinical settings due to its wide array of translational possibilities, sensitivity, and quantitative accuracy. The use of PET has proved to be an instrumental tool in the diagnosis and monitoring of a variety of disease states such as cancer [27]. This review will provide a discussion of recent advances in the development of imaging agents for tumor-associated macrophages focusing specifically on agents for Positron Emission Tomography (PET). For more information about MRI technologies, we refer the reader to the excellent review by Mukherjee et al. [28].

## 2. [^18^F]FDG-PET

In the field of oncology, 2-deoxy-2-[fluorine-18]fluoro-D-glucose (FDG) is a widely used PET imaging probe for the detection and staging of numerous types of cancer. FDG is an analog of glucose which allows for the ability to provide functional information of the glucose metabolism of cancer cells [29].

[^18^F]FDG PET has also been explored in relation to TAMs. In 2015, Cencini and colleagues investigated the relationship between [^18^F]FDG-PET assessment of classical Hodgkin lymphoma (cHL) and TAMs in their roles as potential prognostic predictors. Their study involved the retrospective analysis of 200 cHL patients from 2005–2012. They hypothesized that a positive early [^18^F]FDG-PET assessment could be synonymous with a high TAM score. The patients’ disease progression was staged using a bone marrow biopsy and [^18^F]FDG-PET/CT. Therapy consisted of 2-6 cycles of ABVD (doxorubicin, bleomycin, vinblastine and dacarbazine) [30]. TAMs were determined by immunohistochemistry for CD68, a marker for macrophages. Key results showed that 81.5 percent of patients had a negative [^18^F]FDG-PET which exhibited a positive correlation for progression free survival in that it was significantly higher in [^18^F]FDG-PET negative patients. CD68 expression was classified as low, intermediate, or high and demonstrated no significant difference between the groups when compared with progression free survival. The authors concluded that TAMs did not show a correlation with [^18^F]FDG-PET in classical Hodgkin lymphoma possibly due to the selected TAM markers, antibodies, or cut-off values for TAM scores [30].

More recently, [^18^F]FDG-PET was utilized by Jeong and colleagues to explore the relationship between TAMs with hypoxia and aerobic glycolysis as these have been linked to poor response to chemotherapy and radiotherapy [31,32,33]. This study sought to demonstrate both clinically and preclinically that TAMs contribute to aerobic glycolysis and hypoxia through the competition of oxygen and glucose with cancer cells. Studies were conducted using mice with subcutaneous tumors and patients with non-small cell lung cancer [31]. Patients with stage I and II non-small cell lung cancer were enrolled in the study and 98 biopsy samples were collected: 48 adenocarcinomas and 50 squamous cell carcinomas. Previously, these patients were also imaged with [^18^F]FDG-PET and immunohistochemistry was conducted for TAMs by using CD68 antibodies to differentiate between tumors with low CD68 and high CD68 levels. Contrary to the previous study, significant results showed that there was a strong correlation between [^18^F]FDG uptake and TAMs in tumors. The authors also observed that high CD68 expression was associated with a higher [^18^F]FDG Standardized Uptake Value (SUV). However, when considering the subtype of non-small cell lung cancer (adenocarcinomas or squamous cell carcinomas), [^18^F]FDG uptake and TAM levels only showed a significant correlation in adenocarcinomas. In addition, a strong correlation was found between CD68 and glycolysis related molecules such as GLUT1 and HK2 through the utilization of The Cancer Genome Atlas [31]. The preclinical studies were used to further explore the clinical findings and involved the implantation of LLC tumor cells subcutaneously in mice in order to measure [^18^F]FDG uptake by PET/MRI. Clodronate liposome was also administered intravenously in order to deplete TAMs. Small animal imaging results demonstrated that [^18^F]FDG signals were significantly lowered by the administration of Clodronate liposome [31].

Early in 2020, Ohashi and colleagues explored the relation of M2-macrophage polarization and [^18^F]FDG-PET in head and neck squamous cell carcinoma (HNSCC). Their study involved 73 advanced HNSCC patients in which four parameters were studied using [^18^F]FDG PET/CT: the standardized uptake value, mean standardized uptake value metabolic tumor volume, and total lesion glycolysis. Key results demonstrated that increased glucose uptake, SUVmax, and SUVmean were correlated with higher M2-macrophage polarization [34].

## 3. Imaging Agents for CD206 (Macrophage Mannose Receptor)

The macrophage mannose receptor (CD206 or MMR) is a C-type lectin that is closely associated with the M2 phenotype in tumor-associated macrophages making it a potential biomarker of several types of cancer [35,36]. The development of PET imaging probes that are capable of targeting CD206 have the potential to advance the understanding of the biology of cancer as well as contribute to the discovery of innovative vehicles for the delivery of imaging or therapeutic agents to the tumor microenvironment. Currently, there are several CD206 targeting PET imaging probes that have been studied preclinically.

Locke and colleagues contributed to this area of research in 2012 by conducting a study that involved utilizing PET to image TAMs associated with lung cancer in a mouse model. The imaging agent consisted of a mannosylated liposome (MAN-LIP) that was radiolabeled with copper-64 [37]. Liposomes were chosen due their advantageous characteristics of their lipid bilayer to deliver imaging agents, over previously used molecular imaging agents for TAMs in lung cancer such as nanoparticles attached to small molecules [38,39]. Key results included MAN-LIPs exhibiting a significantly higher association with M2 macrophages versus plain liposomes in in vitro studies and a correlation of lung tumor location with MAN-LIPs signal when compared to remote lung locations in in vivo studies. In addition, the verification of macrophages within the area of lung tumors and internalization of liposomes by TAMs was accomplished by confocal microscopy. They also demonstrated in this study that MAN-LIPS, when compared to PEG liposomes, are quickly cleared from the blood and normal lung [37].

Blykers and colleagues approached targeting CD206 with the utilization of a fluorine-labeled camelid derived single-domain antibody fragment(sdAb) in 2015 [40]. The capability of these antibody fragments to serve as targeting moieties for CD206 positive macrophages has been previously shown [41,42,43,44]. This group developed novel sdAbs that specifically targeted CD206 with a cross reactivity for both mouse and human CD206 through the utilization of an immune sdAb phage-display library. Four potential lead candidates were identified through surface plasmon resonance measurements to expose binders with nanomolar affinity. Flow cytometry revealed only two (anti-MMR 3.49 and 14.4) possessed affinity for cells that expressed human and mouse CD206. The lead compound was further advanced with a biodistribution study of tumor bearing mice with Technetium-99m labeled sdAb which demonstrated rapid renal clearance, and specific retention in the liver, spleen, lymph nodes, bone, and 3LL-R Lewis lung carcinoma tumor cells. In addition, specificity for the anti-MMR 3.49 to CD206 was accomplished through an in vivo study with CD206 deficient mice which demonstrated no tracer uptake excluding the normal route of excretion. Technetium-99m was replaced with Fluorine-18 in order to take advantage of Positron Emission Tomography’s higher sensitivity when compared to Single Photon Emission Computed Tomography (SPECT). In vivo biodistribution illustrated specific targeting of fluorine-18-sdAb to CD206 expressing organs and tissues such as the liver and the TAMs in the tumor. Control mice, which possessed reduced macrophage numbers in the tumor microenvironment, also showed significantly lower tumor uptake when directly compared with wild type mice. PET imaging results agreed with the biodistribution results in that retention in the tumor and CD206 expressing organs such as the liver was apparent. When compared with CD206 deficient mice, this control only showed evidence of uptake in the kidneys and bladder [40].

**Figure 1:**
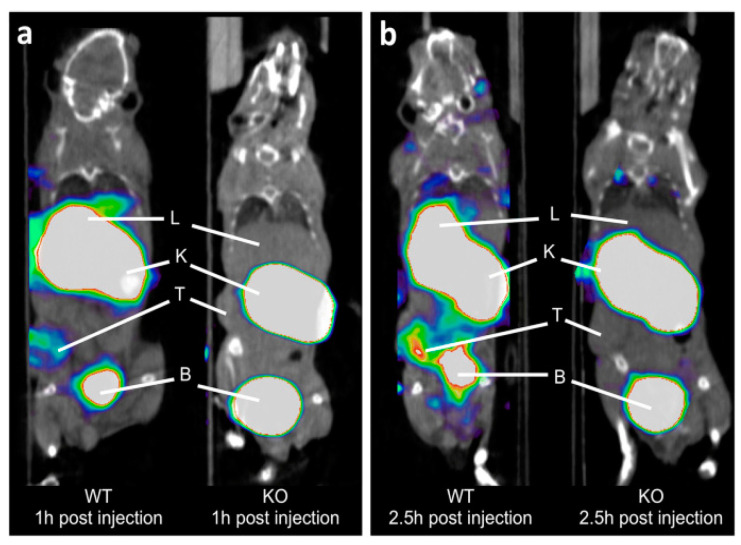
Coronal PET/CT images of [^68^Ga]Ga-NOTA-anti-MMR-sdAb in 3LL-R tumor-bearing wild-type (WT) and CD206-deficient (KO) mice. Images collected at: (**a**) 1 h post injection and (**b**) 2.5 h post injection. PET signals in color scale overlaid on CT image in gray scale. T, K, L, and B are tumor, kidney, liver, and bladder, respectively. From Xavier, C., et al. [45] used with permission.

Xavier and colleagues continued their research with the utilization of single-domain antibody fragments to target CD206 with PET imaging, in hopes of measuring the presence of protumorigenic TAMs, in a recent study published in 2019 [45]. They optimized their previous PET probe from their 2015 study to incorporate gallium-68 instead of fluorine-18 to make it more translatable to clinical evaluation when considering the precedence that Keyaerts and colleagues created with their phase I study of a gallium-68 nanobody for PET/CT assessment of HER2 expression in breast carcinoma [46]. This probe proved to be stable in both the final buffer and in human plasma for 4 h and 1 h, respectively. In addition, the conjugation of the chelating group (NOTA) and complexation with gallium did not affect the affinity. In vivo studies demonstrated uptake of [^68^Ga]Ga-NOTA-anti-MMR-sdAb in organs such as the liver, spleen, and 3LL-R Lewis lung carcinoma tumors which are known to be areas of higher amounts of CD206 TAMs. CD206 deficient mice served as controls and verified specificity as there was no uptake in healthy organs or the 3LL-R tumors. Additionally, it was shown in imaging and biodistribution studies that the probe was rapidly excreted through the kidneys and urine (Figure 1). Dosimetry studies demonstrated that the kidneys and the bladder would receive the highest dose of up to 90 mGy and up to 42 mGy, respectively. Toxicity studies also showed no adverse effects after a 7-day daily injection. This group plans to initiate a phase I clinical trial with [^68^Ga]Ga-NOTA-anti-MMR-sdAb in cancer patients in the near future [45]. 

Venturing outside the theme of this review, it is worth noting the agent Timanocept for the imaging of macrophages. ^99m^Tc-Tilmanocept is an FDA approved SPECT agent indicated for guiding sentinel node biopsy in several different types of tumors such as clinically node negative breast cancer, melanoma, and oral cavity squamous cell carcinoma. The mechanism of action depends on its mannose moieties which bind to CD206. For more information about ^99m^Tc-Tilmanocept, we refer the reader to the excellent review by Cope et al. [47].

## 4. TSPO Translocator Protein

Tumor progression is related to inflammation as a variety of cancer types emerge from sites of irritation and inflammation. The progression of inflammation is known to rely on macrophage infiltration. This has created an interest in the creation of PET tracers that can target macrophages in order to assess inflammation [48,49].

The translocator protein (TSPO) is primarily found on the outer mitochondrial membrane of various different types of cells such as macrophages, neutrophils, and lymphocytes. There have been numerous studies in literature in which TSPO has been used as a target for PET imaging of inflammation [50,51,52]. Although not specifically targeting TAMs, Wu and colleagues carried out a study in 2014 in which they used N, N-Diethyl-2-(2-(4-(2-[^18^F] fluoroethoxy) phenyl)-5, 7-dimethylpyrazolo [1, 5-a] pyrimidin-3-yl) acetamide ([^18^F]-DPA-714), a tracer targeting TSPO, to differentiate inflammation from tumor [48]. They were able to accomplish this by performing PET imaging with this tracer and numerous tumor models (A549, HT29, U87MG, INS-1 and 4T1) and a hind limb muscular inflammation model by injecting oil of turpentine. When compared with the uptake values in inflammatory muscles, [^18^F]-DPA-714 showed significantly lower values for each tumor model which suggested that this tracer could have the potential to separate tumors from other inflammatory diseases using PET [48].

**Figure 2:**
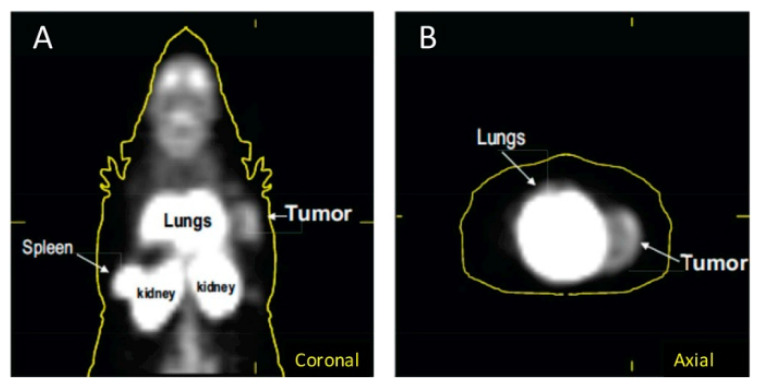
[^11^C]PBR28 uptake in a mouse model of pancreatic ductal adenocarcinoma(PDAC) in (**A**) coronal images and (**B**) axial images. From Lanfranca, M.P., et al. [53] used with permission.

In 2018, Lanfranca and colleagues utilized [^11^C]PBR28, a PET tracer that targets TSPO, in a mouse model of pancreatic ductal adenocarcinoma(PDAC). PDAC is an aggressive type of cancer with a five-year survival rate of less than 10 percent [53]. It has previously been shown in literature that an improved chemotherapy response can be achieved if bone marrow monocyte derived M2 macrophage accumulation to tumors is averted [54]. The aim of this study was to use PET to follow the accumulation and depletion of macrophages. It was demonstrated through autoradiography after tracer injection that there was abundant uptake in the pancreatic tumors when compared to kidney and normal muscle which served as positive and negative controls, respectively. Verification of macrophage specificity for the tracer uptake was also accomplished through autoradiography with the use of a model of macrophage depletion. The mouse model used was Cd11b-DTR which are mice that are genetically modified in myeloid cells to be susceptible to macrophage death when diphtheria toxin (DT) is administered. It was shown that the tracer uptake was significantly less in pancreatic tumors that did not contain macrophages. In vivo data demonstrated uptake in the spleen, kidney, lungs, and readily detected tumor (Figure 2) [53]. 

Sanni and colleagues sought to evaluate N,N-diethyl-2-(2-(4-([^18^F]fluoro)phenyl)-5,7-dimethylpyrazolo[1,5-a]pyrimidin-3-yl)acetamide ([^18^F]F-DPA), another TSPO tracer, in head and neck squamous cell carcinoma (HNSCC) [55]. [^18^F]F-DPA is similar to the tracer discussed previously ([^18^F]-DPA-714) but differs in the placement of the fluorine atom. Nude female mice were injected with either FaDu or Cal33 cells and separated into non-irradiated and irradiated groups. Two experiments were conducted with the FaDu xenografts in which [^18^F]F-DPA and [^18^F]FDG imaging occurred either one or two weeks after radiotherapy. The third experiment was conducted with the Cal33 xenografts in which solely [^18^F]F-DPA imaging was performed one week after radiotherapy. TAM analysis, immunohistochemistry, ex vivo biodistribution, and Western Blot experiments were also executed. Key results demonstrated that [^18^F]F-DPA had a significantly higher uptake in FaDu irradiated tumors when compared to non-irradiated tumors in both one week and two weeks after treatment. [^18^F]FDG uptake in irradiated tumors one week after treatment remained constant whereas uptake was significantly lower in irradiated tumors two weeks after treatment. TSPO expression in tumors showed varied expression with no statistical difference between irradiated and non-irradiated tumors one and two weeks after treatment. Ex vivo biodistribution and autoradiography results also portrayed significantly higher uptake of [^18^F]F-DPA in irradiated tumors versus non-irradiated tumors. Results also demonstrated that radiotherapy decreased the proportion of macrophages in M1 and M2 when compared to non-irradiated tumors but increased non-polarized migratory/macrophages. In addition, TSPO blocking studies done with pre-treatment with PK1195 reduced uptake by 88 percent in non-irradiated tumors and 78 percent in irradiated tumors compared with non-treated cells [55].

Overall, TSPO imaging studies have shown variation in terms of experimental design and aim of the study. TSPO can be useful in differentiating inflammation from tumor which was illustrated through the use of [^18^F]-DPA-714 in numerous tumor models (A549, HT29, U87MG, INS-1 and 4T1) compared to in a muscular inflammation model. Targeting TSPO has also shown promise in tracking the accumulation and depletion of macrophages with [^11^C]PBR28 in a mouse model of pancreatic ductal adenocarcinoma (PDAC). In addition, the effect of radiotherapy on TAMs was investigated with a TSPO targeting agent, [^18^F]F-DPA.

## 5. Endocytosis

Endocytosis is a type of active transport in which materials are engulfed and internalized [56]. Phagocytosis, a subset of endocytosis, mostly occurs in phagocytes such as macrophages. Uptake of materials by macrophages occurs in three main steps which involve the particle reaching the surface of the macrophage, the particle being recognized by receptors on the macrophage membrane, and then changes to the membrane such as protrusion [56]. Endocytic ability of macrophages differs depending on the specific phenotype. The M1 phenotype typically possesses a lower endocytic ability when compared with M2 macrophages [57,58].

The design and application of nanoparticle-based imaging agents exploits this endocytic ability of macrophages. In 2015, Medina and colleagues developed reconstituted high-density lipoprotein nanoparticles radiolabeled with ^89^Zr in order to utilize PET imaging in a breast cancer model [25]. High-density lipoprotein (HDL) has been shown to possess a specificity for macrophages [59,60,61]. The goal of their study was to explore the macrophage targeting capabilities of two radiolabeled HDL-based nanoparticles (^89^Zr-Al-HDL and ^89^Zr-PL-HDL) that differed in the placement of the radiolabel [25]. The overall goal of the design of the tracers was to target TAMs utilizing the biologic function of HDL as opposed to the enhanced permeability and retention effect of passive accumulation. HDL’s main function involves the transport of cholesterol from tissues and cells to the liver [62]. In vivo experiments included pharmacokinetic analysis, biodistribution, and PET imaging. Histologic analysis of tumor tissues was also conducted and flow cytometry to determine cell-targeting specificity. Key results showed that both tracers accumulated in the tumor. Moreover, there was a difference between the blood radioactivity clearance between the two tracers suggesting that HDL’s natural biologic function may be influencing the distribution actively as opposed to solely passively. The biodistribution results showed that most of the radioactivity remains in the blood at 2 h and that tumor uptake increases significantly at the 24-h time point. Kidney, bone, liver, and spleen uptake was also seen at the 24-h time-point, with differences between the two tracers being observed with tumor, kidney and bone. At the 24-h time-point, ^89^Zr-Al-HDL had a significantly higher uptake in the tumor and the kidneys when compared with ^89^Zr-PL-HDL, but demonstrated a significantly lower uptake in the bone. Quantitative PET data coincided with the biodistribution results. Histologic analysis using H&E staining, autoradiography, IBA-1 staining, CD31 staining, and flow cytometry confirmed TAMs as the two tracers’ main target [25].

## 6. Discussion

Tumor-associated macrophages represent a versatile biomarker that have proven to be relevant in the field of oncology and more specifically, in the molecular imaging of cancer biology. The versatility of macrophage imaging in cancer is highlighted with the variability shown in the approaches, designs, and targets of tracers developed. Each tracer discussed demonstrated unique advantages and disadvantages of designing and implementing a macrophage targeting agent.

The utilization of [^18^F]FDG in relation to TAMs has shown variable results. In 2015, Cencini and colleagues concluded that TAMs did not show a correlation with [^18^F]FDG-PET in classical Hodgkin lymphoma despite a 2010 study by Steidl and colleagues that was able to identify a correlation between a high TAM score and results of intervention, also in classical Hodgkin lymphoma [63]. In addition, two studies that also sought to uncover a relationship with TAM score and [^18^F]FDG-PET in classical Hodgkin lymphoma, in 2015, showed conflicting results in that one observed a correlation between the two, while the other did not [64,65]. These differing results were discussed as being possibly due to a variety of reasons such as the selected TAM markers, antibodies for IHC, or cut-off values for TAM scores [30].

It is worth noting recent findings indicating that differentiation of macrophages between the M1 and M2 phenotypes results, at least partially, from a switch in their individual metabolic pathways. Although macrophage metabolism in cancer is not completely understood, key metabolic differences between M1 and M2 macrophages and the relation of metabolism to each phenotype’s specific function, have been reported. One key difference is that M1 macrophages principally rely on aerobic glycolysis for energy which consequently includes an increase in glucose uptake, while M2 macrophages principally rely on fatty acid oxidation and oxidative metabolism for their energy needs [66]. For more information about macrophage metabolism in the context of cancer, we refer the reader to the excellent review by Mehla and Singh [67].

Jeong and colleagues were able to show that there was a strong correlation between [^18^F]FDG uptake and TAMs in non-small cell lung cancer tumors. However, when considering the subtype of non-small cell lung cancer (adenocarcinomas or squamous cell carcinomas), [^18^F]FDG uptake and TAMs only showed a significant correlation in adenocarcinomas [31]. Goodwin and colleagues demonstrated in a 2017 study that squamous cell carcinomas had an increased expression of the GLUT1 glucose transporter which results in a significant glycolysis reliance when compared to adenocarcinomas [68]. The difference in glucose metabolism could explain why there would be a difference in the correlation between [^18^F]FDG uptake and TAMs in the two predominant subtypes of non-small lung cell cancer [31].

The variability of [^18^F]FDG- PET assessment in correlation with TAMs can hint at a possible limitation when considering the use of [^18^F]FDG as means to draw conclusions or observations on TAMs. In addition, [^18^F]FDG-PET also has the limitation of differentiating tumors and non-tumor inflammatory diseases due to its lack of specificity [69]. The utilization of CD206 and TSPO as targets for tumor-associated macrophages could address FDG’s limitations in regard to specificity. However, TSPO has been shown to be present in a variety of cell types so imaging agents targeting it could provide less selective information [70]. CD206 as a PET target for TAMs can provide more specificity but its limitation lies in limited expression of macrophages in diverse phenotypes [71].

Given the importance of TAMs in cancer and advances in macrophage imaging in other disease states, there is an opportunity to employ tracers that have been developed for PET imaging in inflammatory and cardiovascular diseases for imaging in cancer models. For example, the β-isoform of the folate receptor, a glycosylphosphatidylinositol-anchored cell membrane protein, has been shown to be an emerging marker for M2 polarized macrophages, but has not been extensively explored with TAMs. This target has been successfully imaged with [^18^F]fluoro-PEG-folate (polyethylene glycol folate) which was used to visualize rheumatoid arthritis in a first in man study in early 2020 [72]. Future studies may investigate tracers designed for macrophage imaging in other disease states in the cancer setting.

## 7. Conclusions

TAMs have proven their relevance in cancer biology as valuable tools to gain insight on the relationship between immune system response and tumor progression which has resulted in an exciting new era of the development of therapies that target TAMs. Despite an emergence of new immunotherapy techniques, clinical imaging of TAMs has yet to be widely investigated. The imaging of macrophages can provide a noninvasive approach to gain valuable insight into the molecular and functional characteristics of the tumor microenvironment and how the immune response facilitates the progression of cancer or therapy response. The promising data included in this review emphasizes the importance of continuing to expand the research in this area.

## References

[1] Wynn T.A., Chawla A., Pollard J.W. (2013). Macrophage biology in development, homeostasis and disease. Nature.

[2] Gordon S. (2007). The macrophage: Past, present and future. Eur. J. Immunol..

[3] Zhou J., Tang Z., Gao S., Li C., Feng Y., Zhou X. (2020). Tumor-Associated Macrophages: Recent Insights and Therapies. Front. Oncol..

[4] Khan A., Singh V.K., Hunter R.L., Jagannath C. (2019). Macrophage heterogeneity and plasticity in tuberculosis. J. Leukoc. Biol..

[5] Molinaro R., Pastò A., Corbo C., Taraballi F., Giordano F., Martinez J.O., Zhao P., Wang X., Zinger A., Boada C. (2019). Macrophage-derived nanovesicles exert intrinsic anti-inflammatory properties and prolong survival in sepsis through a direct interaction with macrophages. Nanoscale.

[6] Ramdhani S., Navarro E., Udine E., Efthymiou A.G., Schilder B.M., Parks M., Goate A., Raj T. (2020). Tensor decomposition of stimulated monocyte and macrophage gene expression profiles identifies neurodegenerative disease-specific trans-eQTLs. PLoS Genet..

[7] Li Q., Barres B.A. (2018). Microglia and macrophages in brain homeostasis and disease. Nat. Rev. Immunol..

[8] Patel U., Rajasingh S., Samanta S., Cao T., Dawn B., Rajasingh J. (2017). Macrophage polarization in response to epigenetic modifiers during infection and inflammation. Drug Discov. Today.

[9] Zhu X., Tu Y., Chen H., Jackson A.O., Patel V., Yin K. (2018). Micro-environment and intracellular metabolism modulation of adipose tissue macrophage polarization in relation to chronic inflammatory diseases. Diabetes Metab. Res. Rev..

[10] Lin Y., Xu J., Lan H. (2019). Tumor-associated macrophages in tumor metastasis: Biological roles and clinical therapeutic applications. J. Hematol. Oncol..

[11] Chen Y., Song Y., Du W., Gong L., Chang H., Zou Z. (2019). Tumor-associated macrophages: An accomplice in solid tumor progression. J. Biomed. Sci..

[12] Guo Q., Jin Z., Yuan Y., Liu R., Xu T., Wei H., Xu X., He S., Chen S., Shi Z. (2016). New Mechanisms of Tumor-Associated Macrophages on Promoting Tumor Progression: Recent Research Advances and Potential Targets for Tumor Immunotherapy. J. Immunol. Res..

[13] Ardura J.A., Rackov G., Izquierdo E., Alonso V., Gortazar A.R., Escribese M.M. (2019). Targeting Macrophages: Friends or Foes in Disease?. Front. Pharmacol..

[14] Petty A.J., Yang Y. (2017). Tumor-associated macrophages: Implications in cancer immunotherapy. Immunotherapy.

[15] Lee C., Jeong H., Bae Y., Shin K., Kang S., Kim H., Oh J., Bae H. (2019). Targeting of M2-like tumor-associated macrophages with a melittin-based pro-apoptotic peptide. J. Immunother. Cancer.

[16] Allavena P., Sica A., Solinas G., Porta C., Mantovani A. (2008). The inflammatory micro-environment in tumor progression: The role of tumor-associated macrophages. Crit. Rev. Oncol. Hematol..

[17] Belgiovine C., D’Incalci M., Allavena P., Frapolli R. (2016). Tumor-associated macrophages and anti-tumor therapies: Complex links. Cell Mol. Life Sci..

[18] Anfray C., Ummarino A., Andón F.T., Allavena P. (2019). Current Strategies to Target Tumor-Associated-Macrophages to Improve Anti-Tumor Immune Responses. Cells.

[19] Gordon S.R., Maute R.L., Dulken B.W., Hutter G., George B.M., McCracken M.N., Gupta R., Tsai J.M., Sinha R., Corey D. (2017). PD-1 expression by tumour-associated macrophages inhibits phagocytosis and tumour immunity. Nature.

[20] Prasad V., Kaestner V. (2017). Nivolumab and pembrolizumab: Monoclonal antibodies against programmed cell death-1 (PD-1) that are interchangeable. Semin. Oncol..

[21] Lin C.-N., Wang C.-J., Chao Y.-J., Lai M.-D., Shan Y.-S. (2015). The significance of the co-existence of osteopontin and tumor-associated macrophages in gastric cancer progression. BMC Cancer.

[22] Illemann M., Laerum O.D., Hasselby J.P., Thurison T., Høyer-Hansen G., Nielsen H.J., Christensen I.J., for the Danish Study Group on Early Detection of Colorectal Cancer (2014). Urokinase-type plasminogen activator receptor (uPAR) on tumor-associated macrophages is a marker of poor prognosis in colorectal cancer. Cancer Med..

[23] Hu W., Qian Y., Yu F., Liu W., Wu Y., Fang X., Hao W. (2015). Alternatively activated macrophages are associated with metastasis and poor prognosis in prostate adenocarcinoma. Oncol. Lett..

[24] Scott D.W., Steidl C. (2014). The classical Hodgkin lymphoma tumor microenvironment: Macrophages and gene expression-based modeling. Hematology.

[25] Pérez-Medina C., Tang J., Abdel-Atti D., Hogstad B., Merad M., Fisher E.A., Fayad Z.A., Lewis J.S., Mulder W.J., Reiner T. (2015). PET Imaging of Tumor-Associated Macrophages with 89Zr-Labeled High-Density Lipoprotein Nanoparticles. J. Nucl. Med..

[26] Noy R., Pollard J.W. (2014). Tumor-associated macrophages: From mechanisms to therapy. Immunity.

[27] Vaquero J.J., Kinahan P. (2015). Positron Emission Tomography: Current Challenges and Opportunities for Technological Advances in Clinical and Preclinical Imaging Systems. Annu. Rev. Biomed. Eng..

[28] Mukherjee S., Sonanini D., Maurer A., Daldrup-Link H.E. (2019). The yin and yang of imaging tumor associated macrophages with PET and MRI. Theranostics.

[29] Almuhaideb A., Papathanasiou N., Bomanji J. (2011). 18F-FDG PET/CT imaging in oncology. Ann. Saudi Med..

[30] Cencini E., Fabbri A., Rigacci L., Lazzi S., Gini G., Cox M.C., Mancuso S., Abruzzese E., Kovalchuk S., Goteri G. (2017). Evaluation of the prognostic role of tumour-associated macrophages in newly diagnosed classical Hodgkin lymphoma and correlation with early FDG-PET assessment. Hematol. Oncol..

[31] Jeong H., Kim S., Hong B.-J., Lee C.-J., Kim Y.-E., Bok S., Oh J.-M., Gwak S.-H., Yoo M.Y., Lee M.S. (2019). Tumor-Associated Macrophages Enhance Tumor Hypoxia and Aerobic Glycolysis. Cancer Res..

[32] Kroemer G., Pouyssegur J. (2008). Tumor cell metabolism: Cancer’s Achilles’ heel. Cancer Cell.

[33] Brown J.M., Wilson W.R. (2004). Exploiting tumour hypoxia in cancer treatment. Nat. Rev. Cancer.

[34] Ohashi T., Terasawa K., Aoki M., Akazawa T., Shibata H., Kuze B., Asano T., Kato H., Miyazaki T., Matsuo M. (2020). The importance of FDG-PET/CT parameters for the assessment of the immune status in advanced HNSCC. Auris Nasus Larynx.

[35] Fan W., Yang X., Huang F., Tong X., Zhu L., Wang S. (2019). Identification of CD206 as a potential biomarker of cancer stem-like cells and therapeutic agent in liver cancer. Oncol. Lett..

[36] Pei X.-B., Wu X.-Z., Yi F.-S., Zhai K., Shi H.-Z. (2019). Diagnostic value of CD206(+) CD14 (+) macrophages in diagnosis of lung cancer originated malignant pleural effusion. J. Thorac. Dis..

[37] Locke L.W., Mayo M.W., Yoo A.D., Williams M.B., Berr S.S. (2012). PET imaging of tumor associated macrophages using mannose coated 64Cu liposomes. Biomaterials.

[38] Weissleder R., Kelly K., Sun E.Y., Shtatland T., Josephson L. (2005). Cell-specific targeting of nanoparticles by multivalent attachment of small molecules. Nat. Biotechnol..

[39] Melancon M.P., Lu W., Huang Q., Thapa P., Zhou D., Ng C., Li C. (2010). Targeted imaging of tumor-associated M2 macrophages using a macromolecular contrast agent PG-Gd-NIR813. Biomaterials.

[40] Blykers A., Schoonooghe S., Xavier C., D’Hoe K., Laoui D., D’Huyvetter M., Vaneycken I., Cleeren F., Bormans G., Heemskerk J. (2015). PET Imaging of Macrophage Mannose Receptor-Expressing Macrophages in Tumor Stroma Using 18F-Radiolabeled Camelid Single-Domain Antibody Fragments. J. Nucl. Med..

[41] Hamers-Casterman C., Atarhouch T., Muyldermans S., Robinson G., Hammers C., Songa E.B., Bendahman N., Hammers R. (1993). Naturally occurring antibodies devoid of light chains. Nature.

[42] Schoonooghe S., Laoui D., Van Ginderachter J.A., Devoogdt N., Lahoutte T., De Baetselier P., Raes G. (2012). Novel applications of nanobodies for in vivo bio-imaging of inflamed tissues in inflammatory diseases and cancer. Immunobiology.

[43] De Groeve K., Deschacht N., De Koninck C., Caveliers V., Lahoutte T., Devoogdt N., Muyldermans S., De Baetselier P., Raes G. (2010). Nanobodies as tools for in vivo imaging of specific immune cell types. J. Nucl. Med..

[44] Movahedi K., Schoonooghe S., Laoui D., Houbracken I., Waelput W., Breckpot K., Bouwens L., Lahoutte T., De Baetselier P., Raes G. (2012). Nanobody-based targeting of the macrophage mannose receptor for effective in vivo imaging of tumor-associated macrophages. Cancer Res..

[45] Xavier C., Blykers A., Laoui D., Bolli E., Vaneyken I., Bridoux J., Baudhuin H., Raes G., Everaert H., Movahedi K. (2019). Clinical Translation of [68Ga] Ga-NOTA-anti-MMR-sdAb for PET/CT Imaging of Protumorigenic Macrophages. Mol. Imaging Biol..

[46] Keyaerts M., Xavier C., Heemskerk J., Devoogdt N., Everaert H., Ackaert C., Vanhoeij M., Duhoux F.P., Gevaert T., Simon P. (2016). Phase I Study of 68Ga-HER2-Nanobody for PET/CT Assessment of HER2 Expression in Breast Carcinoma. J. Nucl. Med..

[47] Cope F.O., Abbruzzese B., Sanders J., Metz W., Sturms K., Ralph D., Blue M., Zhang J., Bracci P., Bshara W. (2016). The inextricable axis of targeted diagnostic imaging and therapy: An immunological natural history approach. Nucl. Med. Biol..

[48] Wu C., Yue X., Lang L., Kiesewetter D.O., Li F., Zhu Z., Niu G., Chen X. (2014). Longitudinal PET imaging of muscular inflammation using 18F-DPA-714 and 18F-Alfatide II and differentiation with tumors. Theranostics.

[49] Coussens L.M., Werb Z. (2002). Inflammation and cancer. Nature.

[50] Werry E.L., Bright F.M., Piguet O., Ittner L.M., Halliday G.M., Hodges J.R., Kiernan M.C., Loy C.T., Kril J.J., Kassiou M. (2019). Recent Developments in TSPO PET Imaging as A Biomarker of Neuroinflammation in Neurodegenerative Disorders. Int. J. Mol. Sci..

[51] Richards E.M., Zanotti-Fregonara P., Fujita M., Newman L., Farmer C., Ballard E.D., Machado-Vieira R., Yuan P., Niciu M.J., Lyoo C.H. (2018). PET radioligand binding to translocator protein (TSPO) is increased in unmedicated depressed subjects. EJNMMI Res..

[52] Hobson B.A., Rowland D.J., Sisó S., Guignet M.A., Harmany Z.T., Bandara S.B., Saito N., Harvey D.J., Bruun D.A., Garbow J.R. (2019). TSPO PET Using [18F] PBR111 Reveals Persistent Neuroinflammation Following Acute Diisopropylfluorophosphate Intoxication in the Rat. Toxicol. Sci..

[53] Lanfranca M.P., Lazarus J., Shao X., Nathan H., Di Magliano M.P., Zou W., Piert M., Frankel T.L. (2018). Tracking Macrophage Infiltration in a Mouse Model of Pancreatic Cancer with the Positron Emission Tomography Tracer [11C] PBR28. J. Surg. Res..

[54] Sanford D.E., Belt B.A., Panni R.Z., Mayer A., Deshpande A.D., Carpenter D., Mitchem J.B., Plambeck-Suess S.M., Worley L.A., Goetz B.D. (2013). Inflammatory monocyte mobilization decreases patient survival in pancreatic cancer: A role for targeting the CCL2/CCR2 axis. Clin. Cancer Res..

[55] Tuominen S., Keller T., Petruk N., López-Picón F., Eichin D., Löyttyniemi E., Verhassel A., Rajander J., Sandholm J., Tuomela J. (2020). Evaluation of [18F] F-DPA as a target for TSPO in head and neck cancer under normal conditions and after radiotherapy. Eur. J. Nucl. Med. Mol. Imaging.

[56] Ding X., Xiang S. (2018). Endocytosis and human innate immunity. J. Immunol. Sci..

[57] Edin S., Wikberg M.L., Rutegård J., Oldenborg P.A., Palmqvist R. (2013). Phenotypic skewing of macrophages in vitro by secreted factors from colorectal cancer cells. PLoS ONE.

[58] Tarique A.A., Logan J., Thomas E., Holt P.G., Sly P.D., Fantino E. (2015). Phenotypic, functional, and plasticity features of classical and alternatively activated human macrophages. Am. J. Respir. Cell Mol. Biol..

[59] Skajaa T., Cormode D.P., Falk E., Mulder W.J., Fisher E.A., Fayad Z.A. (2010). High-density lipoprotein-based contrast agents for multimodal imaging of atherosclerosis. Arterioscler. Thromb. Vasc. Biol..

[60] Duivenvoorden R., Tang J., Cormode D.P., Mieszawska A.J., Izquierdo-Garcia D., Ozcan C., Otten M.J., Zaidi N., Lobatto M.E., van Rijs S.M. (2014). A statin-loaded reconstituted high-density lipoprotein nanoparticle inhibits atherosclerotic plaque inflammation. Nat. Commun..

[61] Tang J., Lobatto M.E., Hassing L., van der Staay S., van Rijs S.M., Calcagno C., Braza M.S., Baxter S., Fay F., Sanchez-Gaytan B.L. (2015). Inhibiting macrophage proliferation suppresses atherosclerotic plaque inflammation. Sci. Adv..

[62] Fisher E.A., Feig J.E., Hewing B., Hazen S.L., Smith J.D. (2012). High-density lipoprotein function, dysfunction, and reverse cholesterol transport. Arterioscler. Thromb. Vasc. Biol..

[63] Steidl C., Lee T., Shah S.P., Farinha P., Han G., Nayar T., Delaney A., Jones S.J., Iqbal J., Weisenburger D.D. (2010). Tumor-associated macrophages and survival in classic Hodgkin’s lymphoma. N. Engl. J. Med..

[64] Touati M., Delage-Corre M., Monteil J., Abraham J., Moreau S., Remenieras L., Gourin M.P., Dmytruk N., Olivrie A., Turlure P. (2015). CD68-positive tumor-associated macrophages predict unfavorable treatment outcomes in classical Hodgkin lymphoma in correlation with interim fluorodeoxyglucose-positron emission tomography assessment. Leuk. Lymphoma.

[65] Agur A., Amir G., Paltiel O., Klein M., Dann E.J., Goldschmidt H., Goldschmidt N. (2015). CD68 staining correlates with the size of residual mass but not with survival in classical Hodgkin lymphoma. Leuk. Lymphoma.

[66] Galván-Peña S., O’Neill L.A.J. (2014). Metabolic Reprograming in Macrophage Polarization. Front. Immunol..

[67] Mehla K., Singh P.K. (2019). Metabolic Regulation of Macrophage Polarization in Cancer. Trends Cancer.

[68] Goodwin J., Neugent M.L., Lee S.Y., Choe J.H., Choi H., Jenkins D.M.R., Ruthenborg R.J., Robinson M.W., Jeong J.Y., Wake M. (2017). The distinct metabolic phenotype of lung squamous cell carcinoma defines selective vulnerability to glycolytic inhibition. Nature Commun..

[69] Van Waarde A., Cobben D.C., Suurmeijer A.J., Maas B., Vaalburg W., de Vries E.F., Jager P.L., Hoekstra H.J., Elsinga P.H. (2004). Selectivity of 18F-FLT and 18F-FDG for differentiating tumor from inflammation in a rodent model. J. Nucl. Med..

[70] Kreisl W.C., Fujita M., Fujimura Y., Kimura N., Jenko K.J., Kannan P., Hong J., Morse C.L., Zoghbi S.S., Gladding R.L. (2010). Comparison of [(11)C]-(R)-PK 11195 and [(11)C]PBR28, two radioligands for translocator protein (18 kDa) in human and monkey: Implications for positron emission tomographic imaging of this inflammation biomarker. Neuroimage.

[71] Kim H.Y., Li R., Ng T.S.C., Courties G., Rodell C.B., Prytyskach M., Kohler R.H., Pittet M.J., Nahrendorf M., Weissleder R. (2018). Quantitative Imaging of Tumor-Associated Macrophages and Their Response to Therapy Using (64) Cu-Labeled Macrin. ACS Nano.

[72] Verweij N.J.F., Yaqub M., Bruijnen S.T.G., Pieplenbosch S., Ter Wee M.M., Jansen G., Chen Q., Low P.S., Windhorst A.D., Lammertsma A.A. (2020). First in man study of [(18) F]fluoro-PEG-folate PET: A novel macrophage imaging technique to visualize rheumatoid arthritis. Sci. Rep..

